# Hepatic Angiomyolipoma

**DOI:** 10.1155/1994/26843

**Published:** 1994

**Authors:** Mohamad Abid Bakhotmah, Susumo Yamasaki

**Affiliations:** 1 Department of Surgery King Abdulaziz University Hospital Saudi Arabia; 2 Chairman Department of Surgery National Cancer Centre King Abdulaziz University Hospital Saudi Arabia; 3 Assistant Professor King Abdulaziz University Hospital Department of Surgery P.O. Box. 6615 Jeddah 21452 Saudi Arabia

## Abstract

Hepatic angiomyolipomas are rare benign tumours. First reported by Ishak in 1976. Since then, the world literature showed only 32 cases including 8 autopsy findings. In this paper a retrospective analysis of the published data will be presented and the first report ofthe preoperative colour doppler and intraoperative sonography appearances will also be described.


*HPB Surgery*, 1994, Vol. 8, pp. 133–138
Reprints available directly from the publisher
Photocopying permitted by license only

© 1994 Harwood Academic Publishers GmbH
Printed in Malaysia

### CASE REPORT

### Hepatic Angiomyolipoma

MOHAMAD ABID BAKHOTMAH[1] and SUSUMO YAMASAKI[2]

[1]Department of Surgery, [2]Chairman Department of Surgery National Cancer Centre, King Abdulaziz University Hospital

Hepatic angiomyolipomas are rare benign tumours. First reported by Ishak in 1976. Since then, the world literature showed only 32 cases including 8 autopsy findings. In this paper a retrospective analysis of the published data will be presented and the first report of the preoperative colour doppler and intraoperative sonography appearances will also be described.

### CASE REPORT

A 41 year old lady presented to the Out-Patient-Clinic as a case for screening for primary hepatocellular carcinoma (HCC) as she was hepatitis B antigen positive. Her history and physical examination were unremarkable. Blood investigations which included full blood count and liver function tests were all within normal limits. Alpha Feto protein was negative. Hepatic ultrasonography is done as part of the screening procedure, and it was noticed that the lady had a predominantly hyperechogenic mass with areas of hypoechogenic reflections. The mass was 2 × 3 cm and located in segment 6. Although the mass was not typical of early HCC, it was feared that it may be an HCC with fat content, so further assessment with CT, MRI and angiography was requested. The CT scan appearance was that of 2 × 3 cm single mass in segment 6 which was irregular and sharply demarcated, hypodense, partially enhanced in its Left upper corner after injecting contrast material and there was no calcification. The MRI showed a single mass in segment 6 which was of high signal character in both $T_1$, $T_2$ weighted images.

Angiography showed a hypervascular mass but unlike a typical haemangioma. The preoperative color doppler showed in addition to the hyperechogenic mass, areas of vascular sinuses within the tumour. Thus, the preoperative work up suggested a diagnosis of hepatic angiomyolipoma but because (HCC) with fatty metamorphosis cannot be excluded resection of the tumour was carried out in the National Cancer Center, Tokyo. The intraoperative ultrasonography of the liver showed that the mass was located in segment 6 and it was a hyperechogenic mass. Segment 6 resection was performed, the cut section of the mass showed it to be well defined, yellowish in color, 2 × 3 cm in size, with no areas of necrosis and it did not look like an HCC, histology confirmed the diagnosis of angiomyolipoma.

*Address for correspondence*: Mohamad Abid Bakhotmah, Assistant Professor, King Abdulaziz University Hospital, Department of Surgery P.O. Box. 6615, Jeddah 21452, Saudi Arabia

### DISCUSSION

All of the previously reported cases since 1976 until May 1992 was reviewed and analyzed cases in references 2 and 4 were written in Italian and French Languages and the information in the English Summary was not enough for analysis so they were excluded. The number of cases in these reports was 4. Reviewing the previous reported cases was difficult particularly when trying not to include the same cases twice. The aim of this text is to report for the first time; the preoperative color doppler appearance (Fig. 2) and the intraoperative appearance (Fig. 3) of the tumour also we tried to provide a comprehensive text about angiomyolipoma; to avoid extensive details an (*) mark indicates references which can be considered as further reading on that particular subject. After excluding the 4 cases as mentioned previously, the literature contained 27 cases and our case is the 28th. (Table 1) showed the distribution of cases according to various criteria.

In 1976, Ishak described this benign tumour for the first time when he reported the details of 2 autopsy findings[5]. A further 6 autopsy cases were then reported[7,8]. But only in 1983, was the first case diagnosed in a living patient and successfully treated by Kawarada[6]. A retrospective review which contains 14 cases including the previous cases was published by Goodman[8] in 1984; until that time, a total of

**Figure 1** Microscopic appearance of angiomyolipoma notice the fat cell distribution and condensation of the spindle shape muscle cells around the vessels of the tumour. (See color plate I)

**Figure 2** Preoperative color doppler of the angiomyolipoma notice the hyperechoic appearance of the tumour, the 3 vascular sinuses at 6,8,9, o'clock position inside the tumour and the intimate relation of this tumour to the portal vein branch to the affected segment. (See color plate II)

**Figure 3** Intraoperative ultrasonography notice the hyperechoic pattern of the tumour and the posterior echo below the tumour. (See color plate III)

6 cases were diagnosed and 8 cases were autopsy findings. Detailed CT and ultrasonic appearances were described by Takayasu[9] in 1987 followed by first description of the MRI appearance. In 1988, Naito reviewed the world literature[17] and compared the renal and hepatic angiomyolipomas. The ultrastructure appearance was first described by Okada[11]. In 1990, the only fine needle aspirations diagnosis was made pre-operatively[18]. Our report will be the first to our knowledge which showed the preoperative colour doppler and intra-operative ultrasound appearance of this tumour.

This benign rare hepatic tumour seems to be more common in females than males, male:female ratio is 1:1.5; the mean affected age is (50.25 ± 13.58) years. The minimum age was 30 years and the maximum is 76 years and it seems to affect females in a younger age group than males. There is no ethnic group predominance and unlike angiomyolipomas of the kidneys which is associated with tubular sclerosis[17] hepatic angiomyolipomas are not associated with any disease or even specific symptoms, so it is difficult to determine a high risk group for screening. The literature contains no information about parity, drug history etc. Therefore, it is also difficult to accuse any factors like contraceptive pills or female hormones. A screening program for hepatic angiomyolipoma using CT scan can help in understanding the epidemiology of this tumour; but the absence of high risk group makes it difficult.

Pathologically, colour was not described in 6 cases (21.4%), yellow or yellow brown in 7 cases (25%) red or pink in 3 cases (10.7%) and not specified but described as a grade of white to yellow in 12 cases (42.9%).

Described as well demarcated in 26 cases (92.9%) and not well demarcated in 2 cases (7.1%); was not capsulated in 16 cases (57.1%) capsulated in 2 cases (7.1%) partially capsulated in 3 cases (10.7%) and presence or absence of capsule was not mentioned in 7 cases (25%).

So, the gross appearance of the tumour is a round or ovoid well demarcated mass usually yellow in colour and if it is large in size a necrotic area may be seen. Mean size is 7.66 ± 6.03cm mostly located in the right lobe (60.7%).

Microscopically, (Fig 1) as it's name implies, there are 3 components to an angiomyolipoma present together in varying proportions[8*]. The three components are vascular, smooth muscles and fat cells. The tumour is usually well demarcated, but the presence of a fibrous capsule is exceptional.

The vascular component is formed by tortuous vessels, thin walled venous channels or sinuses, and thick walled arteries. This vascular element is found in the form of islands. The fat component is formed by mature adult fat cells forming sheets or individually scattered. The amount of fat present is widely variable from tumour to another.

The smooth muscle component is composed of spindle cell smooth muscle and epithelioid cells in varying proportion but usually spindle shape cells are predominant; when epithelioid cells predominate immunohistochemical and electronmicroscopy will determine its origin. Mitosis was not seen, except rarely in one case which was the largest one[(14)], Haematopoietic elements were also occasionally seen, focal necrosis, nerve cells and focal calcification are rarely demonstrated.

Immunohistochemically[11,12*,13,15,17,18], the muscle component is positive for Desmine stain; vascular endothelial cells are positively stained by the PAP method for factor VIII–related antigen, fat cells are positively stained with sudan-o stain.

Ultrastructure[11*,14,15,18] by electronmicroscopy suggests that the tumour might be derived from certain common predecessor cells located closely to vascular endothelial cells of the liver which are able to accumulate lipid in cytoplasm.

Ultrasound is a sensitive method for detecting angiomyolipomas and it was done in 9 cases (32.1%) [6,8,9,10,13*,16,17]. The appearance is that of hyperechogenic

**Table I** Summary of Published cases of Hepatic Angiomyolipomas

| | *No.of Male Patients* | | *No.of Female Patients* | | *Total% of cases* |
|---|---|---|---|---|---|
| Total No. | Autopsy cases | 6 | Autopsy cases | 2 | 28.6% |
| | Diagnosed cases | 15 | Diagnosed cases | 15 | 71.4% |
| Age Group | 30–45 years | 4 | 30–45 years | 8 | 42.9% |
| | 46–60 years | 3 | 46–61years | 6 | 32.1% |
| | 61–76 years | 4 | 61–76 years | 3 | 25% |
| | mean age 53±16 | | mean age 48±12 | | |
| Affected lobe | Right | 7 | Right | 10 | 60.7% |
| | Left | 2 | Left | 7 | 32.1% |
| | Unknown | 1 | Unknown | 0 | 3.6% |
| | Caudate | 1 | Caudate | 0 | 3.6% |
| Associated Pathology | Aortic Aneurysm | 1 | Asthma | 1 | |
| | Adrenal Adenoma | 1 | Diabetes Mellitus | 1 | |
| | Cerebral Infarct | 1 | Myocrodial Infarct | 1 | |
| | Diabetes Mellitus | 1 | Nephropathy | 1 | |
| | Liver Cihrrosis | 1 | Persistent hepatitis | 1 | |
| | Vellous adenoma | 1 | | | |
| | Nil | 5 | Nil | 12 | |
| Associated Signs & Symptoms | Epigastric pain | 1 | Epigastric pain | 3 | |
| | | | L.U.Q. pain | 1 | |
| | | | R.U.Q. pain | 5 | |
| | | | General fatigue | 2 | |
| | Nil | 10 | Palmar Erythema | 1 | |
| | | | Nil | 5 | |
| Size Range | 1–5 cm | 7 | 1–5 cm | 8 | |
| | 6–10 cm | 1 | 6–10 cm | 4 | |
| | 7–15 cm | 2 | 7–15 | 2 | |
| | 1020 cm | 1 | 10–20 cm | 3 | |

mass sharply demarcated, some are homogenous, others are not; depending on the amount of fat content which varies from 10–50% or even more. Such appearance narrow the differential diagnosis of solid tumour in the liver to, angiomyolipomas, hepatocellular carcinoma with fatty metamorphosis[19], haemangioma and hyperechoic metastasis as from ovarian teratoma; a CT scan is therefore needed for further evaluation.

CT was done in 11 cases (39.3%)[6,8,9,10,12,13*,16,17,18], MRI was done in 2 cases[13*] (7.1%). The appearance is that of a hypodense hepatic mass but unlike metastasis which are usually multiple, irregular and contain calcification, angiomyolipomas contain no calcification and are sharply demarcated, injecting contrast medium to perform CT angiography can differentiate it from an haemangioma; angiomyolipoma partially enhance after infusion of contrast medium because of the fat content. Angiomyolipomas appear as a low attenuation mass but not uniform as in cases of hepatic lipomas. The most important differential diagnosis of angiomyolipoma on a CT scan is lipoma, myolipoma and hepatocellular carcinoma with fatty metamorphosis[19–22]. In the latter case, the fat distribution is rather inhomogeneous and the non-fatty areas pick up the contrast quickly in the early phase of the CT angiography. The MRI appearance is that of high signal well defined mass in both T1–T2 weighted images.

Angiography was done in 7 cases (25%)[6,9,10,12,16,17]. It shows the tumour as a hypervascular mass which is unlike an haemangioma. The radioisotopic appearance is that of positive uptake of TC[99] hyphate, which is identical to focal nodular hyperplasia[16].

The diagnostic method of choice is CT Scan. But it must be remembered that hepatocellular carcinoma can contain fat, so needle biopsy may be useful particularly if it is positive for malignancy.

The preoperative colour doppler (Fig. 2) is like the ultrasound appearance but vascular sinuses can be demonstrated in the tumour prephery.

By intraoperative ultrasound (Fig. 3). The mass appears as a hyperechogenic, well defined mass, it lacks the characteristics of hepatocellular carcinoma; it is not as dense as on haemangioma, and there is no calcification. The mass has the same appearance in all sections of the scan unlike a hepatocellular carcinoma of the same size.

The natural history of the tumour looks like as it is benign, because mitosis or dysplasia are not seen, however, premalignancy cannot be excluded or confirmed.

What to do when one is found? the most difficult question to be answered is whether it is angiomyolipoma or hepatoma with fat metamorphosis, probably resection is the safest approach but if hepatoma can be excluded by fine needle biopsy or by the radiologic appearance follow–up can be recommended.

### ACKNOWLEDGEMENT

We would like to thank the staff of the Liver Surgery Unit in the National Cancer Center for their assistance in the management of the case.

### REFERENCES

1. Meda, Y., Mimura, H., Tsumura. M., *et al.* (1984) A Case of hepatic artery aneurism accompanied with big hepatic angiomyolipoma. *Kanzo*, **25**, 925–930.
2. Liessi, G. Lipoma and angiomyolipoma of the liver. Description of 2 Cases. Radiol. *Med. (Torino)* **5**, 471–3.
3. Shima, Y., Moriyama, N., Takayasu, K., *et al.* (1986) Primary lipoma and angiomyolipoma of the liver diagnosed by CT scan. Report of 2 Resected Cases. *Rinsho Hoshasen* (1986) **31**, 1457–60.
4. Grippon, P., Desaint, B., Herve, de Sigalony. J. P., *et al.* (1985) Angiomyolipoma of the liver. A Fortuitous Echographic Detection. *Gastroenterol Clin Biol.*, **91**, 88–9.
5. Ishak, K. G., Okudu, K., and R. L., Peters, Eds., John Wiley & Sons. (1976) Mesenchymal tumours of the liver in hepatocellular carcinoma. New York 247–307.
6. Kaworada, Y., and Mizumoto, R. (1983) Angiomyolipoma of the liver. *Am, J. Gastroenterol.* **78**, 434–439.
7. Pounder, D. J. (1982) Hepatic, angiomyolipoma. *The American Journal of Surgical Pathology.* **6**, 677–681.
8. Goodman, Z. D., Ishak, K. G. (1984) Angiomyolipomas of the liver. *The American Journal of Surgical Pathology.* **8**, 745–750.
9. Takayasu, K., Sima, Y., Muramatsu, Y., *et al.* (1987) Imaging characteristics of large lipoma and angiomyolipoma of the liver. *Cancer.* **59**, 916–921.
10. Schwartz, P., Travers, P., Hunt, (1989) DR. Angiomyolipoma of the Liver. *Aust N J Surgery.* **59**, 969–971.
11. Okada, K., Yokoyama, S., Nakayama, I., *et al.* (1989) An electron microscopic study of hepatic angiomyolipoma. *The Japanese Society of Pathology.* **39**, (11) 743–749.
12. Angiomyolipoma of the liver. (1991) *Immunohistochemical Study of a Case Liver.* **11**, 158–161.
13. Fobbe, F., Hamm, B. and Schwarting, R. (1988) Angiomyolipoma of the liver, CT, MR and ultrasound imaging. *Journal of Computer Assisted Tomography.* **12**, (14) 658–659.
14. Omohori, T., Arita, N., Uraga, N., *et al.* (1989) Giant hepatic angiomyolipoma. *Histopathology* **15**, 540–3.
15. Weeks, D. A., Malott, R. L., Arnesen, M. *et al.* (1991) Hepatic angiomyolipoma with striated granules and positivity with melanoma-specific antibody (HMB-45). A Report of Two Cases. *Ultrastructural Pathology*, **15**, 563–571.
16. Miyahara, M., Kobayashi, M., Tada, I., *et al.* (1988) Giant hepatic angiomyolipoma simulating focal nodular hyperplasia. *Japanese Journal of Surgery.* **18**, (3) 346–350.
17. Naito, M., Yamamura, F., Takahashi, K., *et al.* (1988) Hepatic angiomyolipoma. A Case Report with Review of Literature. *Acta Pathology Japan* **38**, (6) 799–804.
18. Nguyen, G., Catzavelos, C., Linton, P. L., Ahn, W. S., Schwartz, M. E., *et al.* (1990) Solitary angiomyolipoma of the liver. Report of a Case Initially Examined by Fine Needle Aspiration Biopsy. *Acta Cytologica.* **34**, (2) 201–204.
19. Yoshikawa, Y., Matsui, O., Takashima, T., *et al.* (1988) Fatty metamorphosis in hepato-cellular carcinoma. Radiologic Features in 10 Cases. *AJR* **151**, 717–720.
20. Kaurich, J. D., Coombs, R. J., Zeiss, J. (1988) Myolipoma of the liver. *CT Features Journal of Computed Assisted Tomography.* **12**, (4) 660–661.
21. Itai, Y., Ohtomo, K., Kokubo, T., *et al.* (1987) CT and MR imaging of fatty tumours of the liver. *Journal of Computed Assisted Tomography* **11**, (2) 253–257.
22. Roberst, J. L., Frishman, E. K., Hartman, D. S., *et al.* (1986) Lipomatous tumours of the liver evaluation with CT and U/S radiology. **158**, 613–617.

### INVITED COMMENTARY

Hepatic Angiomyolipoma is a rare benign mesenchymal tumour of the liver, first described by Ishak on two autopsy cases in 1976[1], and first diagnosed before surgery by computed tomography (CT) and successfully treated by a right hepatectomy by Kawarada in 1983[2]. There are about 30 cases reported in the medical literature up to 1992[3].

Histopathologically, angiomyolipoma contains three components, namely blood vessels, smooth muscle and fat, the proportions of which vary between individual tumours and between different parts of the tumour[4]. The vascular component of the tumour consists of tortuous arteries and veins. There are usually numerous thin-walled venous channels or sinuses with variable numbers of conspicuous thick-walled arteries and veins. The thick-walled vessels tended to be more prominent near the periphery of the tumour. The smooth muscle component is usually the most prominent feature, consisting of both spindle and epitheloid cells. The epitheloid cells are polygonal or rounded and are found singly, in clusters or in sheets. A trabecular arrangement was first reported in 1992[3]. The fat component is composed of mature lipocytes in sheets or individual cells scattered throughout the tumour. The proportion of fat can vary from 5 to 90% in different parts within an individual tumour, and vary from less than 10% to over 50% in different tumours[4]. Extrahepatic haemopoiesis is a frequent component in hepatic tumours and an association with tuberous sclerosis is recognised but is a much rarer occurence than it is with renal angiomyolipoma[3,4].

Patients with hepatic angiomyolipoma are either asymptomatic or the symptoms are non-specific. Ultrasound is sensitive in the detection of this tumour because it is echogenic. The ultrasonic appearance is a well defined, smooth contoured and echogenic mass[5]. However, these features are too non-specific to differentiate this tumour from other hepatic pathology such as metastasis or a haemangioma and diagnosis is not easily made using ultrasound examination alone. CT is very sensitive and more specific for hepatic angiomyolipoma, showing it as a low density mass with a fatty attenuation value. Fatty tissue has low attenuation value of usually less than –20 Hounsfield units (HU) on CT on a scale of –1000 for air, 0 for water and +1000 for bone. Kawarada and his associates pointed out that a value of –20 HU is beneficial in diagnosing hepatic angiomyolipoma[2]. The attenuation value, however, depends on the proportion of the fatty component in the tumour. Where the proportion of fat is low within the tumour, the diagnosis may not be suspected unless density measurements are taken at multiple points to confirm the presence of the fat component. In Blumgart's case, density was measured over various sites in the tumour and it ranged from 24.8 HU to –38.7 HU[6]. Angiography is also non-specific and it shows a hypervascular tumour. The main value of angiography is to give a "road map" to the surgeon in planning liver resection. The magnetic resonance imaging appearance has also been reported, showing non-specific high signal and a well defined mass in both $T_1$–$T_2$ weighted images[7]. Hepatic angiomyolipoma takes up technetium 99 m hyphate in much the same way as focal nodular hyperplasia on radionuclide scan[5].

The definitive diagnosis of hepatic angiomyolipoma requires histological confirmation. A list of liver pathology to be differentiated for hepatic angiomyolipoma are primary or secondary liver cancer, haemangioma, focal nodular hyperplasia, adenoma and other rare benign liver tumours. Its diagnosis is made on finding of the three or four components of the tumour, namely blood vessels, smooth muscle, fat and haematopoietic tissue. Percutaneous needle biopsy has the limitation of inadequate tissue sampling although with a high index of awareness of this tumour amongst the pathologist, a definite diagnosis can be reached with needle biopsy[3].

The natural history of liver angiomyolipoma is not yet clear, as few patients have been followed in life for significant periods. In those with symptoms and in those with an uncertain diagnosis, resection has afforded relief of symptoms and confirming the benign nature of the tumour. All patients who have been resected have been cured and there is no evidence of any malignant potential in this tumour.

W.Y. Lau and A.K.C. Li
Prince of Wales Hospital
Shatin, Hong Kong

### REFERENCES

1. Ishak, K. G. (1976) Mesenchymal tumour of the liver. In: Okuda K, Peters K. L., eds. Hepatocellular Carcinoma. New York : John Wiley. 247–304.
2. Kawarada, Y., Mizunoto, R. (1983) Angiomyolipoma of the liver. *Am. J., Gastroenterol*, **78**, 434–439.
3. Tsui, W. M. S., Yuen A. K. T., Ma K. F., Tse C. C. H. (1992) Hepatic angiomyolipomas with a deceptive trabecular pattern and HMB-45 reactivity. *Histopathology*, **21**, 569–573.
4. Goodman, Z. D., Ishak, K. G. (1984) Angiomyolipoma of the liver. *Am, J., Surg. Pathol.*, **8**, 745–750.
5. Miyahara, M., Kobayashi, M., Tada, I., *et al.* (1988) Giant hepatic angiomyolipoma simulating focal nodular hyperplasia. *Jpn. J., Surg.* **18**, 346–350.
6. Blungart, R. L., Payne, M., Rhodes, A. H. (1987) Angiomyolipoma of the liver. *Clin. Radiol.*, **38**, 329–330.
7. Fobbe, F., Hamm, B., Schwarting, R. (1988) Angiomyolipoma of the liver, CT., MR. and ultrasound imaging. *J. Comput. Assist. Tomogr.* **12**, 658–659.

